# Composition and Structure of Aspen (*Pópulus trémula*) Hemicelluloses Obtained by Oxidative Delignification

**DOI:** 10.3390/polym14214521

**Published:** 2022-10-25

**Authors:** Valentina S. Borovkova, Yuriy N. Malyar, Irina G. Sudakova, Anna I. Chudina, Dmitriy V. Zimonin, Andrey M. Skripnikov, Angelina V. Miroshnikova, Vladislav A. Ionin, Alexander S. Kazachenko, Valentin V. Sychev, Ilya S. Ponomarev, Noureddine Issaoui

**Affiliations:** 1 Institute of Chemistry and Chemical Technology, Krasnoyarsk Science Center, Siberian Branch Russian Academy of Sciences, Akademgorodok 50/24, 660036 Krasnoyarsk, Russia; 2 School of Non-Ferrous Metals and Material Science, Siberian Federal University, Pr. Svobodny 79, 660041 Krasnoyarsk, Russia; 3 Department of Biological Chemistry with Courses in Medical, Pharmaceutical and Toxicological Chemistry, Krasnoyarsk State Medical University of the Ministry of Healthcare of the Russian Federation, St. Partizan Zheleznyak, Bld. 1, 660022 Krasnoyarsk, Russia; 4 Laboratory of Quantum and Statistical Physics (LR18ES18), Faculty of Sciences, University of Monastir, Monastir 5079, Tunisia

**Keywords:** hemicellulose, aspen wood, antioxidant activity, delignification, molecular weight characteristics, optimization of the process, HSQC

## Abstract

In this study, hemicelluloses of aspen wood (*Pópulus trémula*) were obtained by oxidative delignification in an acetic acid-water-hydrogen peroxide medium at temperatures of 70–100 °C and a process time of 1–4 h. The maximum polysaccharide yield of up to 9.68 wt% was reported. The composition and structure of the hemicelluloses were studied using a complex of physicochemical methods: gas and gel permeation chromatography, Fourier-transform infrared spectroscopy, 2D nuclear magnetic resonance spectroscopy, and thermogravimetric analysis. The xylose, mannose, galactose, and glucose monomer units were identified in the hemicelluloses by gas chromatography. The weight average molecular weight *M*_w_ of the products determined by gel permeation chromatography was found to range within 8932–33,142 g/mol. The reported Fourier-transform spectra of the hemicelluloses contain all the bands characteristic of heteropolysaccharides; a weak lignin absorption signal in the spectra at 1500–1510 cm^−1^ is attributed to a minor content of phenolic fragments in the structure of the obtained hemicelluloses. The use of thermogravimetric analysis established that the hemicelluloses isolated from aspen wood are resistant against heating to temperatures of up to 90–100 °C and, upon further heating up to 400 °C, start destructing at an increasing rate. The antioxidant activity of the hemicelluloses was examined using the compounds that mimic free radicals (1,1-diphenyl-2-picrylhydrazyl) and hydroxyl radicals (salicylic acid). It was found that the activity of all polysaccharides in neutralizing DPPH and hydroxyl radicals is lower than the absorption capacity of vitamin C at all the tested concentrations (0.5, 2, and 5 mg/mL) and attains 81.7 and 82.9%, respectively.

## 1. Introduction

The use of fossil fuels (crude oil, coal, and natural gas) not only leads to their rapid depletion, but also has a serious negative impact on the environment, in particular, significant emissions of greenhouse gas into the atmosphere [1,2,3]. Recently, there has been a steadily growing trend toward the replacement of fossil fuels by lignocellulosic biomass biofuels. Lignocellulosic biomass has been proven to be an ideal alternative resource, and due to its large amount, short regeneration cycle, biodegradability, and environmental friendliness, can significantly reduce the amount of fossil sources used [4,5,6].

The main components of lignocellulosic biomass are cellulose, hemicellulose, and lignin. Although cellulose is the most widely used polysaccharide [7,8,9,10], hemicellulose, being the second most common renewable natural polymer, remains understudied, but has attracted increasing attention [11] as a potential resource for application in various fields [4,12,13]. Depending on the type of raw material used, hemicelluloses can have different compositions: in deciduous plant species, partially acetylated xylans dominate, while coniferous species consist mainly of glucomannans [14]. The xylan-rich hemicelluloses can be used as hydrogels and biofilms for tissue engineering, pharmaceutical packaging, and drug delivery [15,16], and as adhesives, coatings, stabilizers, and viscosity-inducing agents [16,17].

Due to its compact and rigid structure, lignocellulose is resistant against chemical and biochemical impacts. The fractionation of lignocellulose biomass is affected by several factors [18], but the most serious obstacle to decomposing the lignocellulose polymer components and forming monomeric sugars is considered to be lignin [18,19,20]. The pretreatments increase the porosity of cell walls, thereby making the biomass more amenable to further hydrolysis. In early studies [21,22], various biomass pretreatment methods were used, but few of them have proven their efficiency. A promising pretreatment method is oxidative delignification in an acetic acid-water-hydrogen peroxide medium characterized by a high ability of separating lignin from cellulose and hemicellulose without their degradation and use of high temperatures or strong acids [4,18,23,24,25]. In this delignification process, hydrogen peroxide and acetic acid react with the formation of peracetic acid, which converts the lignin aromatic rings into carboxylic acids by oxidative cleavage [4,25]. In our recent studies, we reported on the method of oxidative delignification pretreatment in an acetic acid–water–hydrogen peroxide medium for further extraction of hemicelluloses from coniferous wood: spruce [4] and larch [26]. In this work, we used hardwood raw materials, specifically, the *Pópulus trémula* aspen wood, which is widely distributed in the boreal and temperate ecosystems of Eurasia [27].

This study is aimed at the optimization of isolation of hemicelluloses (HC) from *Pópulus trémula* aspen wood by oxidative delignification in an acetic acid-water-hydrogen peroxide medium, gel permeation chromatography (GPC) examination of the dependence of the HC molecular weights on delignification conditions, gas chromatography (GC) investigations of the HC monosaccharide composition, exploration of the structural changes and thermal stability of the obtained samples by Fourier-transform infrared (FTIR) and nuclear magnetic resonance (NMR) spectroscopy and thermogravimetric analysis (TGA), and the study of the HC antioxidant activity (AOA) using the compounds that model free and hydroxyl radicals to characterize them for future applications.

## 2. Materials and Methods

### 2.1. Raw Material

The raw material used was air-dry aspen wood (*Pópulus trémula*) grown in the Krasnoyarsk Territory, Russia sampled from the middle part of the stem.

The chemical composition of the absolutely dry aspen wood sawdust (a fraction of ≤2.5 mm) determined by conventional wood chemistry analytical methods [28] included cellulose (48%), lignin (18%), hemicellulose (20%), extractives (11.5%), and minerals (ash, 3%).

### 2.2. Aspen Wood Delignification and Extraction of the Hemicelluloses

**Figure 1:**
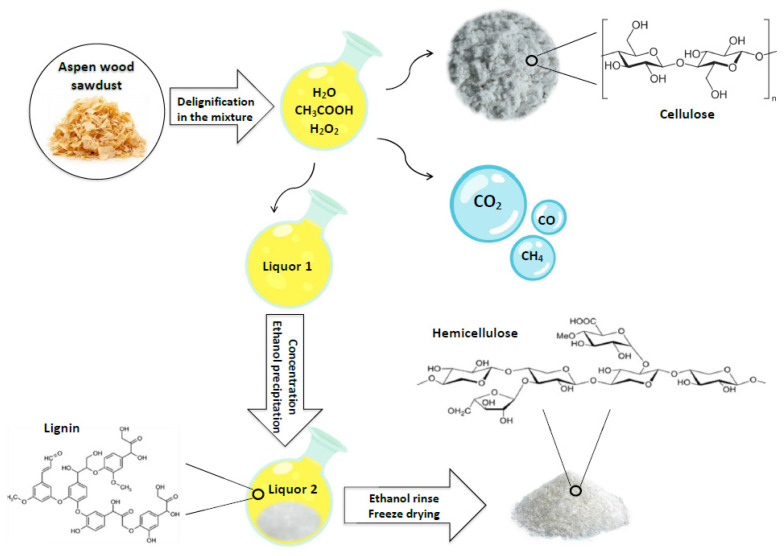
Scheme of oxidative fractionation of aspen wood in the hydrogen acetic acid-water-hydrogen peroxide medium with the formation of cellulose, hemicelluloses, and lignin products.

The scheme of the noncatalytic oxidative fractionation of aspen wood in the acetic acid-water-hydrogen peroxide medium with the formation of cellulose and HCs is shown in Figure 1.

Chopped aspen wood was delignified using a well-proven technique [4,26,29]. The variable parameters of the process are given in Table 1.

The synthesized HCs were analyzed by FTIR and 2D NMR spectroscopy, GC, GPC, and TGA. The AOA was studied using the compounds modeling free radicals (1,1-diphenyl-2-picrylhydrazyl (DPPH)) and hydroxyl radicals (iron(II) sulfate, hydrogen peroxide, and salicylic acid).

### 2.3. Gel Permeation Chromatography

The weight average molecular weight *M*_w_ and polydispersity index PDI of the HC samples were determined by GPC using an Agilent 1260 Infinity II multi-detector GPC/SEC system with a refractive detector. The separation was made on two Agilent PL aquagel-OH columns using the solution of 0.1 M NaNO_3_ with 0.25 g/L NaN_3_ in deionized water as a mobile phase. The column was calibrated using Agilent polyethylene glycol standards (US). The eluent flow rate was 1 mL/min and the sample volume was 100 µL. Before the analysis, the samples were dissolved in the mobile phase (5 mg/mL) and filtered through a 0.45 µm Agilent PES membrane filter (Millipore, Burlington, MA, USA). The data collection and processing were performed using the Agilent GPC/SEC MDS software.

### 2.4. Analysis of the Monosaccharide Composition

To determine the monosaccharide composition, the aspen HCs were hydrolyzed by a 10% H_2_SO_4_ [30] solution for 2.5 h. The individual composition and monosaccharide content in the hydrolysates were determined on a VARIAN-450 GC gas chromatograph equipped with a flame ionization detector on a VF-624ms capillary column with a length of 30 m and an inner diameter of 0.32 mm. The hydrolysate sample was pre-derivatized by the technique described in [31] with the formation of trimethylsilyl derivatives. The silylation reagent used was a mixture of trimethylchlorosilane and hexamethyldisilazane in pyridine; sorbitol was used as an internal standard (IS). Standards for analyzing the hydrolysates were glucose, arabinose, galactose, sorbitol, mannose, and xylose (Panreac, Darmstadt, Germany).

### 2.5. Fourier-Transform Infra-Red Spectroscopy

The IR spectra were recorded on a Shimadzu IRTracer-100 FTIR spectrometer (Shimadzu Co., Kyoto, Japan). Specimens for recording the IR absorption spectra were pressed in tablets containing 3 mg of the sample in a potassium bromide matrix.

### 2.6. Nuclear Magnetic Resonance

The NMR analysis (heteronuclear single quantum coherence (HSQC) spectroscopy) of HCs was made on a Bruker Avance III 600 spectrometer (Bruker, Rheinsetten, Germany). Before the analysis, the sample was completely dissolved in the DMSO-d6 solvent and placed into a 5 mm NMR tube at room temperature.

### 2.7. Thermogravimetric Analysis

The TGA study was carried out on a NETZSCH STA 449 F1 Jupiter simultaneous thermal analysis instrument (Selb, Germany). The HC samples were analyzed in argon at a heating rate of 10 °C min^−1^, temperatures from 30 to 900 °C, and protective and blowout gas flow rates of 20 and 50 mL min^−1^, respectively. The Al_2_O_3_ cylindrical crucible with a perforated cover was used and a reference was an empty corundum crucible with a cover. The instrument was calibrated according to the specification using reference substances supplied with the instrument. The sample weight for the analysis was determined on a Sartorius BP121S analytical lab scale digital balance. The measurement data were processed using the NETZSCH. Proteus Thermal Analysis.5.1.0 software supplied with the instrument.

### 2.8. Optimization

The mathematical processing was performed in the Statgraphics Centurion XVI application software package, DOE (Design of Experiment) block. The experiment plan was combined multi-level (Users Design).

### 2.9. Antioxidant Activity

#### 2.9.1. DPPH Radical Scavenging Assay

The absorption capacity of DPPH was used to establish the HC antioxidant activity determined by the method described in [4,32]. A solution of DPPH in ethanol (0.2 mmol/L) was prepared before the UV measurements. The HC samples were dissolved in distilled water in concentrations of 0.5, 2, and 5 mg/mL. The polysaccharide solutions (1 mL) were thoroughly mixed with 2 mL of freshly prepared DPPH and 2 mL of ethanol. The mixtures were well-mixed and kept at room temperature for 30 min in the dark. After that, the absorbance was measured at 517 nm against a blank. The low absorption of the reaction mixture is indicative of the high free radical scavenging activity, as can be seen in the inhibition percent versus compound concentration plot. In this study, vitamin C (Vc) was used as a positive control. The experiments were repeated three times and the values obtained were averaged.

The scavenging ability of DPPH was calculated as shown in Equation (1):

(1) $$\mathrm{DPPH}\;\mathrm{Radical}\;\mathrm{Scavenging}\;\mathrm{Ability}\;(\%)=\left(1-\frac{A_{S}-A_{B}}{A_{C}}\right)*100\%,$$

where A_C_ is the absorbance of the DPPH solution without a sample, A_S_ is the absorbance of the test sample mixed with the DPPH solution, and A_B_ is the absorbance of the sample without DPPH solution.

#### 2.9.2. Hydroxyl Radical Scavenging Assay

The hydroxyl radical assay was measured by the method described in [32]. With some modifications in this experiment, HCs were dissolved in deionized water in concentrations of 0.5, 2, and 5 mg/mL. The sample solution (2 mL) was mixed with 2 mL of 6 mM ferrous sulfate and 2 mL of 6 mM H_2_O_2_. After standing for 10 min, 2 mL of 6 mM salicylic acid was added, mixed thoroughly, and kept standing for more 30 min. The absorbance of the mixture was measured at 510 nm against blank. The capability to scavenge the hydroxyl radical was calculated using the Equation (2):

(2) $$\mathrm{Hydroxyl}\;\mathrm{Radical}\;\mathrm{Scavenging}\;\mathrm{Ability}\;(\%)=\left(1-\frac{A_{S}-A_{B}}{A_{C}}\right)*100\%,$$

where A_C_ is the absorbance of the solution without a sample, A_S_ is the absorbance of the test sample mixed with the reaction solution, and A_B_ is the absorbance of the sample without salicylic acid solution.

## 3. Results

### 3.1. Hemicellulose Delignification and Yield

Hemicelluloses were extracted from the liquid products of the noncatalytic oxidative delignification in the acetic acid-water-hydrogen peroxide medium after isolation of cellulose. Peracetic acid formed in the reaction of hydrogen peroxide with acetic acid is a powerful oxidizing agent, which leads to the significant oxidative degradation and ensures the removal of lignin from the lignocellulose matrix [33,34]. 

The data on the effect of temperature and time of the delignification process on the yield of HCs extracted from the products of the noncatalytic oxidative delignification of aspen wood in the acetic acid-water-hydrogen peroxide medium are given in Table 2.

It was found that the HC yield at process temperatures of 70–90 °C is lower than at 100 °C. This is probably due to the fact that, at 100 °C, delignification of wood is more complete and a larger HC amount passes into the solution. Importantly, at a process temperature of 100 °C, over the entire range of process times (1–4 h), the highest product yield is observed and, with an increase in the hydrolysis time, the product yield grows. Thus, the high temperature and longtime facilitate the HC hydrolysis reactions.

### 3.2. Optimization of the Oxidative Delignification

The effect of the main process factors on the oxidative delignification of aspen wood was examined. Two factors included in the study as independent variables were reaction temperature and time. The process temperature varied at three levels (80, 90, and 100 °C) and the time varied at two levels (3 and 4 h). The result of the process was characterized by two output parameters: HC weight fraction in the product and polydispersity. The designations of the variables are given in Table 3.

The results of experiments on oxidative delignification of aspen wood used in the mathematical processing and optimization are listed in Table 4.

The statistical characteristics are given in Table 5.

The analysis of variance showed that, under the accepted experimental conditions, both the temperature and time of delignification contribute significantly to the total variance of the output parameter.

The dependence of the HC yield on the process variables was approximated by the second-order regression Equation (3):

*Y*_1_ = 78.879 − 1.861 *X*_1_ − 5.878 *X*_2_ + 0.0106 *X*_1_^2^ + 0.0855 *X*_1_*X*_2_. (3)

**Figure 2:**
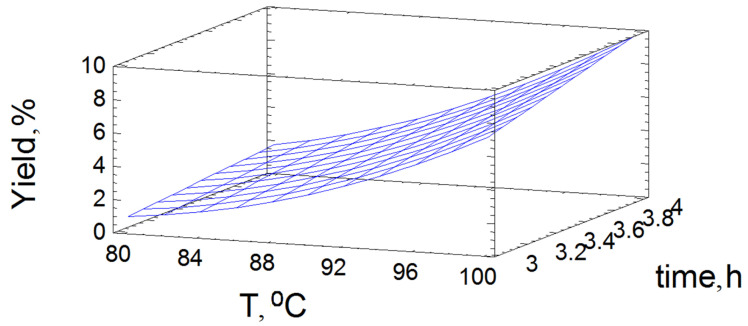
Response surface of the output parameters under different impacts of the experimental conditions on the HC yield.

The adequacy of Equation (3) is confirmed by the high determination coefficient *R*^2^_adj_ = 99.6%. The mathematical model used to build the response surface clearly illustrates the effect of the time and temperature of oxidative delignification of aspen wood on the product yield (Figure 2). 

**Figure 3:**
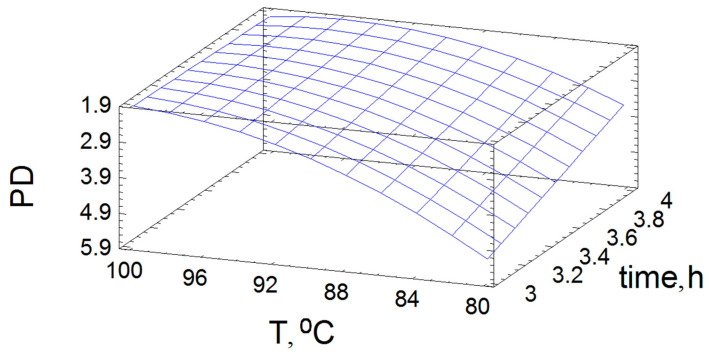
Response surface of the output parameters under different impacts of the experimental conditions on the polydispersity index.

The maximum predicted value (9.567%) in the investigated area of the factor space is obtained, according to the calculation using mathematical model (Figure 3), at the point corresponding to a process temperature of *T* = 100 °C and a process time of 4 h. As can be seen in Figure 1, the response surface is almost flat with a strong slope toward higher temperatures and a minor slope toward longer process times, which is consistent with the data on the analysis of variance. This shape of the response surface points out the intensification in the destruction of the lignocarbohydrate complex with the increasing time of oxidative delignification of wood.

Similarly, the optimum conditions for *Y*_2_ were calculated. The dependence of *Y*_2_ on the process variables is approximated by the second-order regression Equation (4):

*Y*_2_ = 98.2 − 1.626 *X*_1_ − 8.99 *X*_2_ + 0.0066 *X*_1_^2^ + 0.091 *X*_1_
*X*_2_. (4)

Using the mathematical model, the dependence of the PDI on the variable factors T and time was presented graphically in the form of a response surface (Figure 3). 

The minimum PDI is obtained at the point with a process temperature of T = 98.8 °C and a process time of 3.51 h. 

### 3.3. Gel Permeation Chromatography Study of the Hemicelluloses

To illustrate the dependence for the degree of depolymerization of the noncatalytic oxidative delignification of aspen wood in the acetic acid-water-hydrogen peroxide medium on the process temperature, the molecular weight characteristics of the HC samples obtained at the longest process time (4 h) in the entire temperature range (70–100 °C) were determined by GPC using a refractometric detector.

The obtained molecular weight characteristics for the aspen wood HC samples are given in Table 6.

It was established that the HC samples isolated from the liquid products of delignification under the acidic conditions have an average molecular weight of *M*_w_ = 8932–33,142 g/mol and a PDI of 2.050–6.175. According to the data obtained, with a decrease in the process temperature, the molecular weights significantly increase, probably due to the incompleteness of delignification, i.e., separation of HCs from other high-molecular cellulose products, as well as to a large amount of monosaccharides and residual lignin fragments in the composition of the HCs isolated under these conditions. Therefore, the molecules have a cross-linked branched structure. The branching and heterogeneity of molecules that increase with a decrease in the process temperature is indicated also by the increasing PDI values.

**Figure 4:**
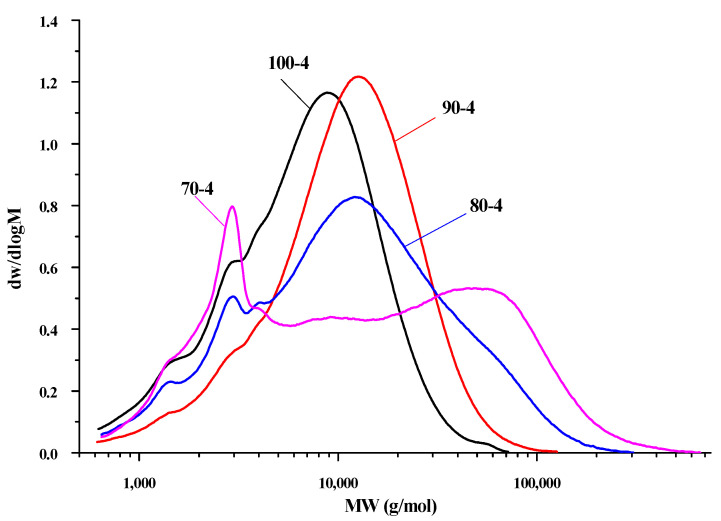
Curves of the molecular weight distribution of the aspen wood hemicelluloses obtained at 4 h and different temperatures.

According to the molecular weight distribution curves (Figure 4), all the samples have a response in the low-molecular-weight region (~5000 g/mol), which probably includes difficult-to-separate lignin fractions and, at lower temperatures (70–80 °C), oxidative delignification of aspen is low-efficiency, which was established by the GPC study.

However, in this experiment, in sample 100-4 with the highest yield of the main product (9.68 wt%), the appearance of signals in the low-molecular-weight region can be explained by the fact that, with increasing temperature, long oligomer chains are hydrolyzed and the insoluble HC fractions start destructing, until a sufficiently small length is reached at which dissolution occurs without further hydrolysis. On the other hand, if the process is sufficiently long (4 h), the HCs that could be extracted at 100 °C was probably already dissolved and their molecular weight starts slowly decreasing for several possible reasons: (i) hydrolysis starts flowing not only in the entire system, but specifically within the HC structure; (ii) C–C bonds in the HC structure are broken, and (iii) functional groups are cleaved from side chains and, consequently, low-molecular-weight compounds are isolated. All these factors point out that, from the beginning to the end of the process, HCs are subjected to continuous hydrolysis.

Since the highest yields were achieved over the entire process time range (1–4 h) at 100 °C, we decided to estimate the degree of depolymerization of samples 100-3, 100-2, and 100-1 (Table 7).

At the beginning of the depolymerization process (1 and 2 h) at 100 °C, molecular weights of up to 10,484 g/mol were obtained, but, as the extraction progresses, the molecular weight increases and attains its maximum of *M_w_* = 11,228 g/mol in 3 h (sample 100-3).

**Figure 5:**
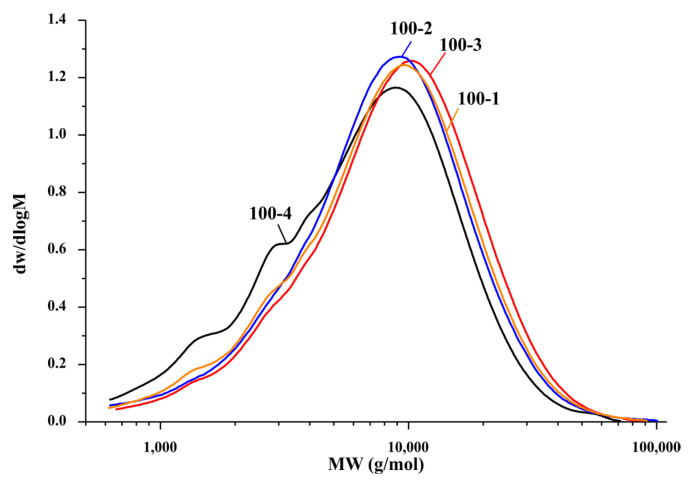
Curves of the molecular weight distribution of the aspen wood hemicelluloses obtained at 100 °C.

According to the molecular weight distribution curves (Figure 5), the HCs isolated in the noncatalytic delignification process with times of 1, 2, and 3 h at 100 °C have similar MWD profiles and are fairly homogeneous.

In contrast to sample 100-4, samples 100-3–100-1 have no distinct signals in the low-molecular-weight region; i.e., the amount of HC that passes into solution is larger than the amount of other lignocellulosic products and the depolymerization process proceeds with a sufficiently high efficiency without degradation of the HC structure or precipitation of low-molecular compounds.

The resulting HCs with a high degree of depolymerization (samples 100-4, 100-3, 100-2, and 100-1) were analyzed using FTIR spectroscopy, 2D NMR, GC, and TGA methods.

### 3.4. Analysis of the Monosaccharide Composition of the Hemicelluloses

**Figure 6:**
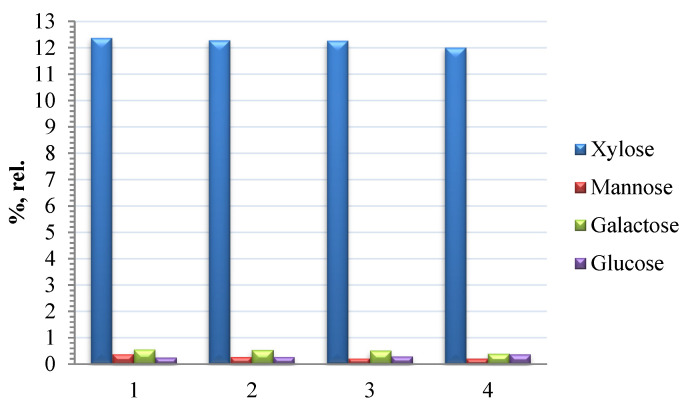
Monosaccharide composition of the hemicelluloses for samples (1) 100-4, (2) 100-3, (3) 100-2, and (4) 100-1 isolated from the liquid products of oxidative delignification of aspen wood (percentage of the total monosaccharide content).

The carbohydrate composition of the HCs obtained by the noncatalytic oxidative delignification of aspen wood is shown in Figure 6.

The GC study of the hydrolyzate composition revealed xylose, mannose, galactose, and glucose. The composition of the monosaccharides and, above all, the dominance of xylose, indicate that, under the process conditions used, the easily hydrolyzable polysaccharides are subjected to hydrolysis [35]. Glucose, which is contained in the hydrolyzate in a small amount, is probably the product of the hydrolysis of HCs and the amorphous part of cellulose.

The high xylose content in the mixture of the aspen HC monosaccharides indicates that glucuronoxylan is the leading polysaccharide in the obtained HCs, which is typical of hardwood HCs [36,37]. The presence of mannose, galactose, and glucose is indicative of glucomannan, arabinogalactan, and other polysaccharides in small amounts [38].

### 3.5. Fourier-Transform Infra-Red Spectroscopy

**Figure 7:**
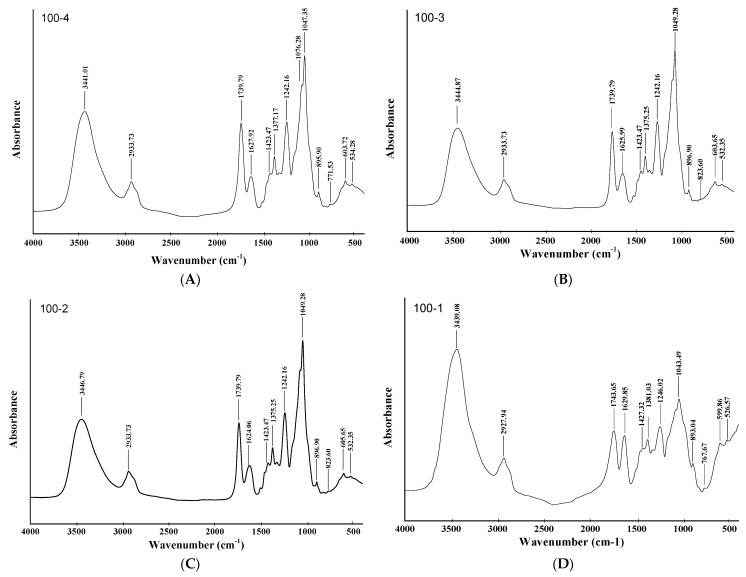
IR spectra of the aspen hemicelluloses and lignin moieties: 100-4 (**A**), 100-3 (**B**), 100-2 (**C**) and 100-1 (**D**).

The FTIR spectra of the HC samples contain all the bands characteristic of polysaccharides. In focus were the fundamental frequencies of the absorption bands of xylan as the main aspen HC component. The FTIR spectra of the HC samples are presented in Figure 7. In these spectra, several regions can be distinguished [39,40,41].

In particular, the hydroxyl groups included in the intermolecular H bond have a characteristic absorption band in the range of 3460–3100 cm^−1^.

The band at about 2935 cm^−1^ characterizes the asymmetric CH_2_ vibration. A separate band at ~2853 cm^−1^ corresponds to the symmetric stretching vibration of the methylene group. In the region of ~1735 cm^−1^, there are absorption bands of the C=O bond in uronic acids. The region 1650–1620 cm^−1^ is characterized by the absorption of water due to the strong affinity of water for xylan.

The symmetric bending vibrations of CH_2_ appear around 1430 cm^−1^. Bands of asymmetric vibrations of the CH_3_ groups lie in the same region. The absorption at 1380 cm^−1^ is related to the bending vibrations of the end CH_3_ in the acyl groups. Meanwhile, the absorption band at about 1240 cm^−1^ corresponds to the CO bond in the acetyl group, which reflects acetylation of the aspen wood HCs. The band at 1045 cm^−1^ is attributed to the C–O–C stretching vibration of the xylan bond [40]. The band at ~900 cm^−1^ in the xylan spectrum characterizes the configuration at the first carbon atom of the pyranose ring (β-configuration at about 891 cm^−1^ and α-configuration at 844 cm^−1^) [41]. The out-of-plane OH bending vibrations of the interaction between xylan units lie between 770–500 cm^−1^. In addition, the HC samples obtained at 100 °C for different process times contain a minor number of phenolic fragments, as evidenced by a weak signal of the absorption band in the region of 1500–1510 cm^−1^.

### 3.6. Nuclear Magnetic Resonance

**Figure 8:**
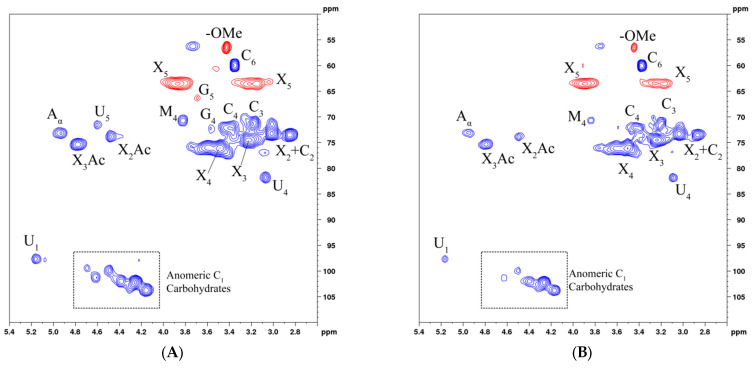

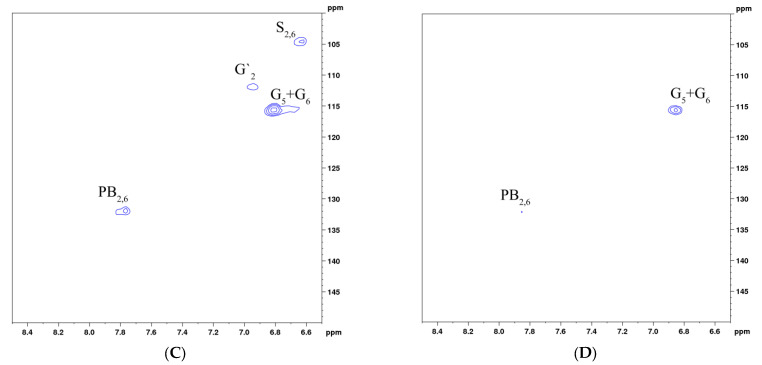
HSQC spectrum of the aspen hemicelluloses, samples 100-1 (**A**,**C**) and 100-4 (**B**,**D**): aliphatic oxygenated region (**A**,**B**) and aromatic region (**C**,**D**).

The HSQC spectrum of the aspen hemicelluloses are presented in Figure 8.

The δH/δC spectral range of 2.7–3.9/70–85 contains the signals characteristic of atoms C2–C4 of mannose, galactose, glucose, and xylose. In the δH/δC spectral range of 3.05–4.0/55–65, there are signals from carbohydrate atoms C5 and C6 [42]. The δH/δC cross peaks related to the mannose, glucose, galactose, and xylose molecules were observed at 4.60–4.70/100.0–103.0, 4.50/99.0, 4.35/103.0, and 4.1–4.3/102.0–105.0 ppm, respectively. The cross signals observed in the spectra showed the signals corresponding to (1→4)-β-D-XylT of the bonds (X2: 2.8/73.0, X3: 3.2/74.0, X4: 3.45/76.0, X5: 3.8/63.5, and 3.0–3.35/63.5), as well as the signals 4-OMe-α-d-Glcp (3.8/71.0) and the signals (1→4)-β-d-Xylp-2-O-(4-OMe-α-d-GlcpA) of the bonds (3.4/73.5) and residue (1→3)-α-l-Ara (3.5/61.5) [43]. 

Obviously, the identified δH/δC cross peaks point out that β-d-Xylp is a backbone of the aspen wood HCs, which contain a linear xylan backbone linked by the β-(1→4) glycosidic bonds.

We have to mention the disappearance of signals U5 (4.6/71.6), G4 (3.55/72.3), and G5 (3.68/66.4)), as well as a decrease in the intensity of the signals in the aromatic region (Figure 8C,D) of the HSQC spectrum of the HCs with an increase in the oxidative delignification time. This is evidence for the cleavage of residual lignin units and an increase in the HC purity [44]. 

### 3.7. Thermogravimetric Analysis

**Figure 9:**
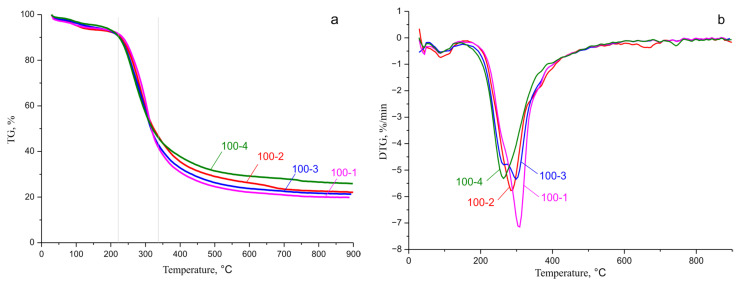
Thermogravimetry curves: (**a**) TG; (**b**) DTG for the aspen hemicellulose samples.

The thermal properties of the HC samples were studied by the TGA method. Figure 9a,b shows thermogravimetry (TG) and derivative thermogravimetric (DTG) curves for the aspen HC samples.

The hemicelluloses extracted from aspen wood are resistant against heating up to about 90–100 °C and, upon further heating, start destructing at an increasing rate. The thermal decomposition of the HC samples can be divided into three stages. 

At the first stage, the weight loss is observed up to 90–100 °C, which is caused by the evaporation of adsorbed water. At the second stage, the intensive HC decomposition occurs, which begins at ~220 °C for all the samples; at relatively low (up to 180–220 °C) temperatures, reactions of partial deacetylation of HCs (hydrolysis of ester groups) seem to dominate and reactions of hydrolytic degradation of polysaccharides occur due to the formation of acetic acid. When the temperature rises above 220 °C, the thermal destruction reactions already occur with the hemolytic opening of glycosidic and C–C bonds in monosaccharide units. In the investigated HC samples, the most rapid weight loss was observed at 230–320 °C. The intensive decomposition ends at ~400 °C. Importantly, the second phase of the thermal decomposition of HCs is accompanied by the destruction of C–C and C–O bonds associated with the main HC branch, as well as decarboxylation and decarbonylation, which lead to the high reactivity of HCs with the high decomposition pyrogenicity. At this stage, the HC polymers decompose with the release of CO, CO_2_, and some light hydrocarbons, including CH_4_ and C_2_H_4_ [45]. At the last stage, when the temperature exceeds 390 and 420 °C, the weight loss in the samples is negligible and the removal of volatile compounds lasts until the temperature reaches 899.8 °C.

### 3.8. Analysis of the Antioxidant Activity of the Hemicelluloses

#### 3.8.1. Scavenging Activity of the DPPH Radicals

**Figure 10:**
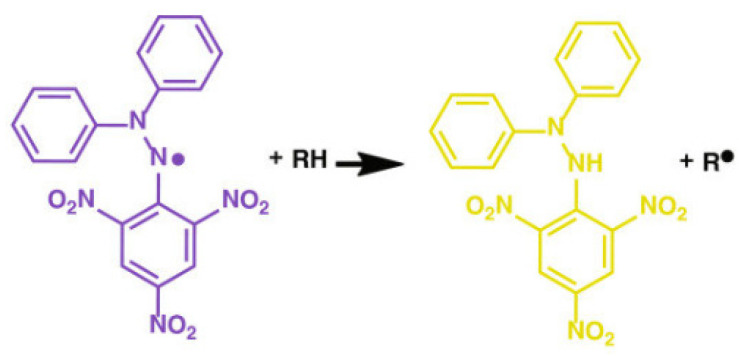
Scheme of the stable diphenylpicrylhydrazine formation resulting from the interaction of the antioxidant with DPPH [47].

It was shown in many studies [4,32,46] that plant polysaccharides are able to scavenge free radicals and can be used as natural antioxidants. To determine the ability of antioxidants to scavenge free radicals, DPPH is commonly used, showing the strong absorption at 517 nm [4]. The antioxidant can couple with one DPPH electron with the formation of stable yellow diphenylpicrylhydrazine (DPPH-H) (Figure 10) with a decrease in the absorption with the increasing antioxidant molecule concentration.

**Figure 11:**
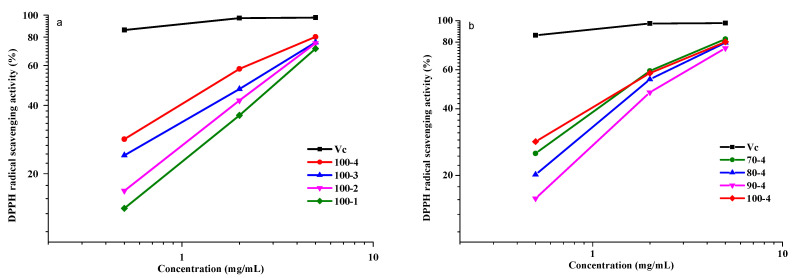
DPPH radical scavenging activity. (**a**) HCs obtained at 100 °C and Vc and (**b**) HCs obtained at 4 h and Vc, at different concentrations.

This study showed that the aspen HCs have a strong inhibitory effect on the DPPH radicals; in this case, a dose-dependent pattern is observed (Figure 11). However, the hydrolysis conditions can significantly affect the DPPH radical scavenging. As can be seen in Figure 11a, sample 100-4 has the stronger DPPH radical scavenging effect at all concentrations (up to 81.7%), in contrast to samples 100-3–100-1 obtained at shorter process times. Sample 100-1 showed the minimum effect on the removal of the DPPH radicals (69.1%) over the entire concentration range. This result demonstrated that the HC extraction process time significantly affects the ability of HCs to absorb DPPH.

The content of carboxyl and methoxy groups of uronic acids, as well as the high content of hydroxyl groups, affect positively the AOA of polysaccharides [47]. The uronic acids formed in the long-term hydrolysis apparently make a great contribution to the AOA of sample 100-4, which is confirmed by the consistency of the GPC and AOA data. In addition, the positive effect of the galactose content in polysaccharides on the AOA was reported [4], which is consistent with the data on the monosaccharide composition: the highest galactose content is observed in sample 100-4. According to [48,49], the polysaccharides with a relatively low molecular weight show a stronger ability to absorb DPPH radicals. It is important to note, however, that, although sample 70-4 has the highest molecular weight (33,142 g/mol), its AOA is similar to that of sample 100-4. The factors that can determine the high AOA of sample 70-4 are the lignin fractions (phenolic component) unseparated because of the low process temperature (70 °C) and cellulose fragments, the presence of which also has a positive effect in terms of the AOA.

#### 3.8.2. Scavenging Activity of the Hydroxyl Radicals

**Figure 12:**
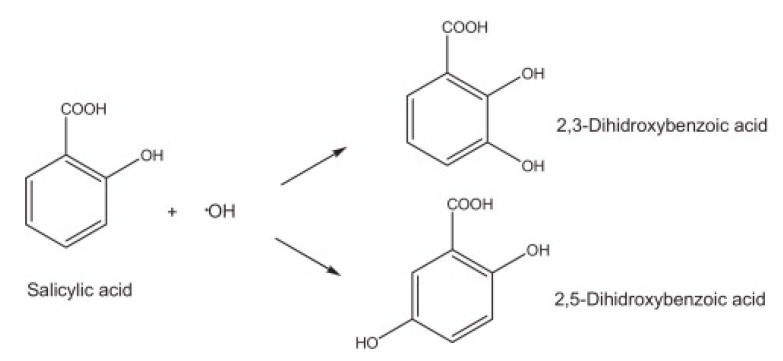
Reaction of salicylic acid with a hydroxyl radical [50].

Salicylic acid can react with the hydroxyl radicals with the formation of 2,3-dihydroxybenzoic or 2,5-dihydroxybenzoic acid (Figure 12) [50].

**Figure 13:**
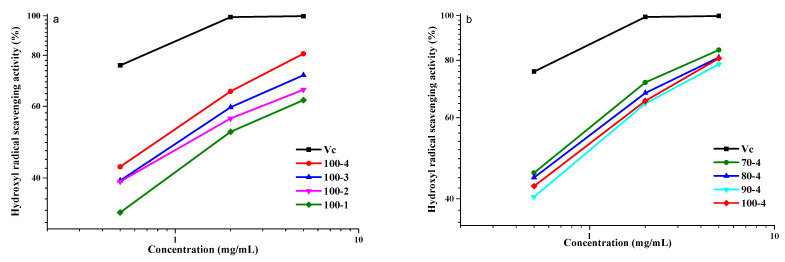
Hydroxyl radicals scavenging activity: (**a**) HCs obtained at 100 °C and Vc and (**b**) HCs obtained at 4 h and Vc, at different concentrations.

These two isomers exhibit the UV absorption at 510 nm [32,50]. The stronger the absorption, the stronger salicylic acid binds to the OH radicals. Vc or polysaccharides can also scavenge the hydroxyl radicals (Figure 13).

Similar to paragraph 3.8.2, Vc was used as a positive control. As shown in Figure 13, the HCs isolated from aspen wood over the entire concentration range (0.5–5.0 mg/mL) showed the ability to remove •OH. Samples 70-4 and 80-4 (82.9 and 81.9%, respectively) exhibited the higher AOA than sample 100-4. As is known, the phenolic compounds are capable of removing radicals. The more active sample, 70-4, has a higher content of associated phenolic components (residual lignin), according to the GPC data, which may contribute to the effect of the removal of the OH radicals. 

The uronic acid content can also affect the ability to remove OH radicals. Sample 100-4 containing a greater amount of uronic acid exhibited the much stronger AOA than samples 100-3–100-1, which apparently contain less uronic acid [51].

## 4. Conclusions

The noncatalytic oxidative delignification of aspen wood in the acetic acid-water-hydrogen peroxide medium was studied. The maximum HC yield (up to 9.68 wt%) was obtained at a process temperature of 100 °C and a process time of 4 h.

Using the mathematical processing, the optimum conditions for delignification of aspen wood were determined: a process temperature of 98.8 °C and a process time of 3.51 h. 

It was established that the aspen wood HCs isolated under the chosen conditions have different molecular weight distributions and weight average molecular weights (8932–33,142 g/mol). With an increase in the process temperature from 70 to 100 °C, the PDI decreases from 6.18 to 2.05, indicating an increase in the homogeneity of the HC.

The isolated HCs with a high degree of depolymerization consist mainly of glucuronoxylan and contain a small amount of residual lignin (the weak signal in the lignin absorption band in the FTIR spectra at 1500–1510 cm^−1^). It was confirmed by the HSQC method that β-d-Xylp is the basis of aspen wood HCs, which contain a linear xylan backbone linked by glycosidic bonds β-(1→4) with minor amounts of hexose, mannose, and uronic acid units in the side chains.

The HCs isolated from aspen wood are resistant against heating up to temperatures of ~90–100 °C; the most rapid weight loss was observed at 230–320 °C. The intense decomposition ends at a temperature of ~400 °C for all the samples.

The activity of all polysaccharides in neutralizing the DPPH and hydroxyl radicals was lower than the absorption capacity of vitamin C at all the tested concentrations (0.5, 2, and 5 mg/mL). It was found that the polysaccharides with a low molecular weight and a high content of uronic acids had a stronger ability to donate hydrogen. Samples 70-4 and 80-4 containing more phenolic components exhibited the stronger ability to remove hydroxyl radicals; however, in terms of purity of the main product, they are inferior to samples 100-4–100-1.

## Figures and Tables

**Table 1:** Variable parameters of oxidative delignification of aspen wood (“+” is conducting an experiment, “-“ is not conducting an experiment).

Process Temperatures (°C)	70	80	90	100
Process Times (h)				
**4**	+	+	+	+
**3**	-	+	+	+
**2**	-	-	-	+
**1**	-	-	-	+

**Table 2:** Hemicellulose yield from the soluble products of the noncatalytic oxidative delignification of aspen wood at process temperatures of 70–100 °C and times of 1–4 h.

Process Temperatures (°C)	70	80	90	100
Process Times (h)	Hemicellulose Yield (wt%) ^1^			
**4**	0.67 ^2^	1.81 ^3^	4.79 ^5^	9.68 ^8^
**3**	-	0.92 ^4^	2.83 ^6^	7.08 ^9^
**2**	-	-	1.09 ^7^	6.30 ^10^
**1**	-	-	-	4.92 ^11^

^1^ Relative to the initial air-dry aspen wood. ^2^ Hemicelluloses (70-4) obtained by noncatalytic delignification at a process temperature of 70 °C and a process time of 4 h. ^3^ Hemicelluloses (80-4) obtained by noncatalytic delignification at a process temperature of 80 °C and a process time of 4 h. ^4^ Hemicelluloses (80-3) obtained by noncatalytic delignification at a process temperature of 80 °C and a process time of 3 h. ^5^ Hemicelluloses (90-4) obtained by noncatalytic delignification at a process temperature of 90 °C and a process time of 4 h. ^6^ Hemicelluloses (90-3) obtained by noncatalytic delignification at a process temperature of 90 °C and a process time of 3 h. ^7^ Hemicelluloses (90-2) obtained by noncatalytic delignification at a process temperature of 90 °C and a process time of 2 h. ^8^ Hemicelluloses (100-4) obtained by noncatalytic delignification at a process temperature of 100 °C and a process time of 4 h. ^9^ Hemicelluloses (100-3) obtained by noncatalytic delignification at a process temperature of 100 °C and a process time of 3 h. ^10^ Hemicelluloses (100-2) obtained by noncatalytic delignification at a process temperature of 100 °C and a process time of 2 h. ^11^ Hemicelluloses (100-1) obtained by noncatalytic delignification at a process temperature of 100 °C and a process time of 1 h.

**Table 3:** Designations of independent variables (factors) and output parameters (results of experiments).

Factors and Parameters	Designations	
	In the Text and Figures	In the Equations
Temperature (°C)	T	*X*_1_
Time (h)	t	*X*_2_
Hemicellulose yield (%)	Yield	*Y*_1_
Polydispersity	PD	*Y*_2_

**Table 4:** Effect of experimental conditions on the characteristics of the aspen hemicelluloses.

Sample	Reaction Temperature (°C)	Reaction Time (h)	Characteristics	
			Yield, Mass %	PDI
-	*X*_1_ (T)	*X*_2_ (t)	*Y*_1_	*Y*_2_
80-3	80	3	0.973	5.22
90-3	90	3	2.833	2.94
100-3	100	3	7.075	1.92
80-4	80	4	1.812	3.53
90-4	90	4	4.785	2.10
100-4	100	4	9.678	2.05

**Table 5:** Data on the analysis of variance.

Variance Sources	Output Parameters			
	Yield *Y*_1_, Mass %		Polydispersity, *Y*_2_	
	Statistical Characteristics			
	Variance Relations *F*	Significance Levels *p*	Variance Relations *F*	Significance Levels *p*
*X*_1_: T	1096.8	0.0113	4760.1	0.0092
*X*_2_: Prod	160.6	0.0355	800.0	0.0225
*X*_1_^2^	48.8	0.0642	484.0	0.0289
*X*_1_ *X*_2_	23.7	0.0918	690.1	0.0242
Number of degrees of freedom	2		2	
*R* ^2^_adj_	99.6		99.9	

**Table 6:** Molecular weight characteristics of the aspen wood hemicelluloses obtained at 4 h and different temperatures.

Sample	*M_w_* (g/mol)	PDI
70-4	33,142	6.175
80-4	20,266	3.528
90-4	14,149	2.102
100-4	8932	2.050

**Table 7:** Molecular weight characteristics of the aspen wood hemicelluloses obtained at 100 °C.

Sample	*M_w_* (g/mol)	PDI
100-4	8932	2.050
100-3	11,228	1.918
100-2	10,290	1.926
100-1	10,484	1.988

## Data Availability

All data generated during this study are included in the article.
